# Barriers faced by primary healthcare providers in addressing emergencies in the Northern region of Palestine before and during the Gaza war

**DOI:** 10.1186/s12875-024-02512-3

**Published:** 2024-07-17

**Authors:** Suha Hamshari, Sondos Hamadneh, Mukaram Ghneem, Raghad Khalaf, Sara Daqqa, Rola Abu Alwafa, Mahfouz Ktaifan

**Affiliations:** 1 Department of Medicine, Faculty of Medicine and Health Sciences, An-Najah National University, Nablus, 44839 Palestine; 2 Department of Family Medicine and Community Medicine, An-Najah National University, Nablus, 44839 Palestine; 3 Department of General Surgery, An-Najah National University Hospital, Nablus, 44839 Palestine

**Keywords:** Primary health care, Emergencies, Developing countries, Physicians

## Abstract

**Introduction:**

Physicians working in primary health care (PHC) centers are the first contact for patients and expected to deal with emergencies. Emergency care training in PHC settings has been neglected globally, especially in low to middle income countries. Adequate preparation becomes especially important during periods of conflict. The study objectives are to identify the barriers facing PHC physicians when dealing with emergency cases in the northern region of Palestine during the current conflict.

**Methods:**

A cross-sectional study was conducted with 179 physicians working in the 10 PHC centers distributed among 5 northern governances in Palestine. The study period was from July through December 2023. Data were collected electronically using a self-administered questionnaire, which was adapted from a comprehensive literature review. The questionnaire’s internal validity was confirmed by a Cronbach’s alpha coefficient of [0.85], indicating high reliability.

**Results:**

The ages of the physicians ranged from 25 to 60 years, with a mean ± SD of 35.3 ± 8.15 years. A significant majority (91.6%) were not boarded in any specialty. Most physicians (85.5%) had attended Basic Life Support (BLS) courses, whereas 45.2% and 72% had never attended Advanced Cardiovascular Life Support (ACLS) or Advanced Trauma Life Support (ATLS) courses, respectively. Physicians with emergency department experience (*P* = 0.002) and those who had attended ACLS courses (*P* < 0.001) reported significantly higher perceived competence in managing emergency cases.

**Conclusion:**

Emergency services at PHC centers in northern Palestine are operational but require significant enhancements. There is a critical need for increased availability of essential equipment, supplies, and medications. Additionally, implementing comprehensive training programs in emergency management, particularly ACLS, is essential to improve the competence and performance of PHC physicians in emergency situations.

## Introduction

### Background

A medical emergency is defined as a critical and abrupt situation that necessitates quick action due to the potential life-threatening effects [1]. Workers in primary care settings around the world may encounter numerous emergencies such as asthma, anaphylaxis, shock, dyspnea, seizure, and cardiac arrest [2–4]. Hence, primary care doctors should be prepared to administer basic and advanced life support in situations where there may be a delay in ambulance arrival, or when patients underestimate the urgency of their condition [5].

With the ongoing war in Gaza and the increased atrocities in the West Bank, it is more crucial than ever for primary care physicians to have the skills to handle emergencies. From October 7th to the end of 2023, there was a 50% increase in lethal attacks in the West Bank compared to the first nine months of the year [5]. As an occupied territory, checkpoints and unexpected road closures make travel difficult. This has grown worse on the West Bank with the Gaza war as the Israeli army attacks cars, storms homes, and Settlers are armed [5]. Ambulances are often obstructed from moving and medics have been wounded [5]. More medical emergencies are present to local clinics. Unfortunately, primary care centers and physicians are not well-equipped to handle the emergencies.

Globally, emergency care in primary healthcare settings has been neglected, especially in countries with low or middle incomes like Palestine [6]. Primary health care physicians face many obstacles when dealing with emergency cases including lack of appropriate equipment and medications, unavailability of equipped ambulances to transport cases, and limited competence to respond to emergencies due to limited exposure to cases and the challenges of keeping skills updated [7]. Courses like basic life support (BLS) and advanced cardiac life support (ACLS) can assist in better emergency management [8].

Going back to literature; the current state of emergency care in PHC settings varies globally, due to disparities in resources, infrastructure, and access to training programs specially in low- and middle-income countries. In Palestine specifically, the ongoing conflict in addition to limited resources and infrastructure, exacerbate the challenges of emergency care training in PHC settings. Until now no previous studies have been done in Palestine to study the level of training among PHC workers and factors that influence how to deal with emergencies which can help policymakers to improve emergency care training and strengthen healthcare systems in the region.

Efforts to enhance emergency care training at the PHC level have been done worldwide, but till now, there are many gaps in access to training programs, education and the presence of standardized protocol for dealing with emergencies.

According to a study done in South Africa, The World Health Organization (WHO) advocates for the enhancement of emergency care capabilities at a PHC level as a way of lessening the burden of disease on the overall health system [9].

According to literature; the impact of inadequate emergency training in PHC can increase morbidity and mortality. Many studies conducted in conflict-affected regions to study the maternal and child health in conflict settings documented higher rates of maternal mortality and birth complications due to delay in accessing emergency care or lack of trained healthcare providers in PHC settings. Also the coverage for antenatal care, institutional delivery, and childhood vaccines were low [10, 11].

A few studies made an assessment for trauma care in resource-limited settings revealed that; there is insufficient training in PHC settings on the management of traumatic injuries which may be associated with higher morbidity and mortality rates among trauma patients [12]. To date, no research has been done on the emergency services offered by Palestinian primary health care centers. The study objectives are to identify the barriers facing primary health care physicians when dealing with emergencies in the northern areas of Palestine.

Enhancing emergency preparedness in PHC settings can prompt policymakers to formulate and implement policies aimed at prioritizing emergency response. These policies can include various aspects such as staff training, resource allocation, the process of role and task allocation and the development of protocols for effective emergency cases management, including triage and communication with emergency services.

## Methodology

### Study design and setting

Using a cross-sectional study design, self-administered online questionnaires, were provided electronically to 150 physicians during their work hours at primary health care clinics in the region. An electronic version of the questionnaire was also distributed to 29 primary health care clinicians at home who had difficulty accessing their clinical setting due to the war situation in the west bank .

Data were collected from 10 major PHC centers distributed in 5 geographical areas including Tulkarem, Nablus, Jenin, Salfit, and Qalqilya over a 4-month period.

### Target population, inclusion, and exclusion criteria

Physicians working in primary health care clinics in Northern areas of Palestine were recruited according to the following:

- Inclusion criteria: primary health care physicians providing curative, preventative, and promotional health care services.
- Exclusion criteria: primary health care physicians who do only administrative work and have no contact with patients.

### Sample size and sampling technique

The sample size was determined using Raosoft software. The number of primary healthcare (PHC) physicians in the Northern districts was 510, as indicated by he Palestinian annual health report [13]. The minimal sample size was 220, with a 95% confidence level and a 5% margin of error. A non-probability sampling technique, specifically convenient sampling, was employed. In order to obtain the desired sample for the purpose of study. The samples were collected from physicians working in primary healthcare centers from 10 major PHC sites.

### Data tool

- Part 1 : Socio-demographic data : Age, Gender, Marital status, Residency.
- Part 2 : six questions to identify the level of training in primary health care, experience in primary care, previous working experience in the emergency department (ED) setting, and participation in emergency courses.
- Part 3 : self-reported frequency of emergency cases in the last 12 months.
- Part 4 : questions to detrerimen the perceived level of competence when dealing with eight emergency cases ,
- Part 5 : four main questions about the availability in their settings of equipment, drugs, ambulance, and other supporting facilities that are needed to deal with emergency cases based on other studies [3, 13].
- Part 6 : their satisfaction with emergency services provided at their primary health care centers [3] Appendix 1.

### Validity and reliability of the tool

We pre-tested the questionnaire to ensure validity and reliability. For content internal validity, three experts in the field reviewed the questionnaire and they edited the questionnaire until we have the final version (Appendix 1). Cronbach alpha was calculated for the questionnaire 0.85.

### Data analysis plan

Data entry and analysis were conducted using SPSS software version 25. Mean & standard deviation were used to describe age. Physicians’ characteristics (age, gender, the city where their primary health care centers are located), the frequency of emergency cases, the level of competence to perform emergency skills, the availability of tools, and the physician’s level of satisfaction regarding the management of certain emergency cases were described with frequencies and percentages. A Chi-square test was used to indicate statistical significance at p value less the 0.05 for attending emergency courses, work experience in emergency departments or primary health care clinics, and the perceived level of competence to handle emergency cases.

### Ethical consideration

All procedures performed in this study, involving human participants, were consistent with the research ethical standards. The An-Najah National University Institutional Review Board (IRB) provided ethical approval (Ref: Med. May.2023/24). The Ministry of Health authorized the study at its primary care centers and participants were approached and invited voluntarily to participate. All participants gave an informed consent and the participants were approached and invited voluntarily to participate. Participants were assured of their confidentiality and anonymity.

## Results

There were 179 physician participants. Table 1 shows their **s**ocio-demographic characteristics. Ages ranged between 25 and 60 years with a mean ± SD of (35.3 ± 8.15) years. Almost half of physicians (58.7%) were between 25 and 35 years. Male physicians comprised 56.4% of the sample. The majority of physicians (91.6%) are general practitioners, which means they completed medical school and a one-year rotating hospital-based internship. Physicians boarded in family medicine comprised 5.6% of the sample.

**Table 1:** Socio-demographic characteristics of the primary health care physician respondents

Variables	Frequency (%) or median [Q1-Q3] *n* = 179
**Age**	33 [29–40]
**Gender**	
Male	101 (56.4)
Female	78 (43.6)
**Marital status**	
Married	125 (69.8)
Single	52 (29.1)
Divorced	2 (1.1)
**Job Title**	
General Practice ^a^	164 (91.6)
Family medicine Specialist	10 (5.6)
Gynecologist	1 (0.6)
Pediatrician	4 (2.2)
**City**	
Nablus	55 (30.7)
Jenin	46 (25.7)
Tulkarem	43 (24)
Qalqilya	28 (15.6)
Salfit	7 (3.9)

^a^ General Practice are physicians who completed medical school and a one-year hospital-based internship

Table 2 shows the perceived level of competence to perform emergency skills. Almost half of primary health care physicians (48%) will perform simple suturing and nebulization and oxygen therapy (44.7%). Over a third of the physicians will attempt bag and mask resuscitation (40.2%), cardiac compression (35.8%) and read ECGs (34.1%). One fifth (20.1%) said that “they don’t know where to start” regarding performing defibrillation, whereas (46.9%) said they will intervene if there was no one else available to defibrillate.

**Table 2:** Physicians’ perceived level of competence to perform emergency skills

Emergency Skills	Frequency (%) *n* = 179			
	I do not know where to start	I will do only if no one else is available	I will attempt in most cases	I will attempt in all cases
**Cardiac compression**	1 (0.6)	40 (22.3)	74 (41.3)	64 (35.8)
**Bag & mask resuscitation**	2 (1.1)	32 (17.9)	73 (40.8)	72 (40.2)
**Nebulization & oxygen therapy**	2 (1.1)	24 (13.4)	73 (40.8)	80 (44.7)
**Inserting IV cannula**	8 (4.5)	61 (34.1)	58 (32.4)	52 (29.1)
**Urinary catheter insertion**	9 (5)	68 (38)	58 (32.4)	44 (24.6)
**Reading ECG**	4 (2.2)	33 (18.4)	81 (45.3)	61 (34.1)
**Defibrillation**	36 (20.1)	84 (46.9)	38 (21.2)	21 (11.7)
**Simple suture**	5 (2.8)	31 (17.3)	57 (31.8)	86 (48)

Table 3 compares the perceived level of competence in performing emergency procedures by demographics, experience, and training factors. Males, physicians who had worked in the ED more than one year, and physicians who had taken a BLS and/or ACLS, and/or ATLS course reported significantly more competence. Table 4 shows the frequency of emergency cases seen in primary health care clinics in the last year. Renal colic was the most common, with half of the primary healthcare physicians (50.3%) having seen three or more cases in the last 12 months. Asthma, angina, and hypoglycemia were reported by a third of the physicians as having seen three or more cases in the last 12 months (38%, 32.4%, and 31.9%, respectively). Approximately one quarter (24.6%) have seen three or more cases of acute myocardial infarction, and 8.4% have seen three or more cases of cardiac arrest in the past year.

**Table 3:** Factors associated with perceived level of competence in performing emergency skill scale among primary health care physicians

Variable	**Score of level of competence in performing emergency skill scale (8–32)**			
	Median [Q1-Q3] *n* = 179	*Mean rank*	*OR and CI*	*P*-value ^a^
**Age**				
25–34	18.5 [14.5-23.25]	86.5	0.35 [-0.48-1.21]	0.545 ^c^
35–44	23 [13–24]	96.32		
≥ 45	16 [14.5–19]	91.35		
**Gender**				
Male	16 [13–20]	101.69	2.5 [1.25–3.75]	**0.001** ^**b**^
Female	15 [13–17]	74.87		
**Marital status**				
Married	20 [16–24]	92.01	-0,06[-1.42-1.3]	0.332 ^c^
Single	18.5 [14–23]	90.02		
Divorced	-	36.75		
**Job title**				
GP	18.5 [14.5–23.5]	88.68		
Family medicine	-	102	0.63 [-0.62-1.90]	0.709 ^c^
Gynecologist	-	100		
Pediatrician	16.5 [14–19]	111.63		
**Working years in PHC**				
< 1 year	23.5 [17.75-24]	101.51	-0.53 [-1.41-0.33]	0.199 ^c^
1–5 years	20 [14-23.5]	82.34		
> 5 years	17 [14–21]	90.96		
**Working years in ED**				
< 1 year	15 [12.75–20.25]	82.33	1.44 [0.44–2.44]	**0.008** ^**c**^
1–5 years	19 [16.25–22.75]	95.53		
> 5 years	23.5 [16.75-24]	127		
**BLS Course**				
Yes	16 [13–19]	93.03	0.66 [-1.23-2.55]	**0.057** ^**b**^
No	14.5 [12.5–17]	72.17		
**Duration since attending Basic Life Support**				
<1 year	16 [14.5–24]	74.36	0.24 [-3.65-4.13]	0.830 ^c^
1–2 years > 2 years	23 [18–24]	79.85		
> 2 years	17 [13–19]	76.11		
**ACLS**				
Yes	16 [13.75–20.25]	101.32	1.23 [-1.4-2.61]	**0.001** ^**b**^
No	15 [13–17]	76.3		
**Duration since attending ACLS**				
< 1 year	23 [15.25-24]	59.22	-1.5 [-5.44-2.40]	0.316 ^c^
1–2 years	17 [15–23]	48.67		
> 2 years	18 [13–21]	47.16		
**ATLS**				
Yes	17 [13-21.25]	104.32	1.12 [-0.29-2.54]	**0.021** ^**b**^
No	15 [13–18]	84.45		
**Duration since attending ATLS**				
< 1 year	16.5 [13–23]	25.19	-0.87 [-3.52 -1.76]	0.498 ^c^
1–2 years	21 [15 -22.5]	30.61		
> 2 years	16 [13.5–19.5]	24.18		

^a^ The bold values indicate *p* < 0.05

^b^ Statistical significance values calculated using the Kruskal–Wallis test

^c^ Statistical significance values calculated using the Mann–Whitney U test

**Table 4:** Frequency of self-reported emergency cases seen by primary health care physicians in the last 12 months

Emergency Cases	Frequency (%) *n* = 179		
	None	1–2	≥ 3
Acute asthma	30 (16.8)	81 (45.2)	68 (38)
Myocardial infarction	62 (34.6)	73 (40.8)	44 (24.6)
Angina pectoris	47 (26.3)	74 (41.3)	58 (32.4)
Cardiac arrest	116 (64.8)	48 (26.8)	15 (8.4)
Severe dehydration	61 (34.1)	75 (41.9)	43 (24)
Renal colic	13 (7.3)	76 (42.5)	90 (50.3)
Acute GI bleeding	109 (60.9)	48 (26.8)	22 (12.3)
Hypoglycemia	43 (24)	79 (44.1)	57 (31.9)
Diabetic ketoacidosis	75 (41.9)	74 (41.3)	30 (16.8)
Convulsion	70 (39.1)	77 (43)	32 (17.9)
Anaphylaxis	81 (45.3)	68 (38)	30 (16.7)
Acute vaginal bleeding	115 (64.2)	41 (22.9)	23 (12.8)

Table 5 describes the primary health care center’s readiness to manage emergency care. Cannulas, oxygen masks, dressing trays, and scissors were available in almost all primary health care clinics. On the other hand, defibrillators, splints, suction devices, and endotracheal tubes were only available in (20.7%), (22.9%), (34.6%) and (38%) of primary health care clinics, respectively. Laboratories were available in (90.5%) of clinics, whereas X-ray and equipped ambulances were present in (28.5%) and (6.1%), respectively.

**Table 5:** Availability of equipment, drugs and supporting facilities needed for emergency care at primary health care centers

Item	Frequency (%) *n* = 179
**Equipment**	
Cannula	177 (98.9)
IV stand	160 (89.4)
Urinary catheter	91 (50.8)
Dressing tray	161 (89.9)
Side lamp with stand	112 (62.6)
Splint	41 (22.9)
Scissors	161 (89.9)
Suture kit	157 (87.7)
Ambu bag	109 (60.9)
Nebulizer	159 (88.8)
O2 cylinder with standard fit	137 (76.5)
Laryngoscope	73 (40.8)
Endotracheal tube	68 (38.0)
O2 mask	162 (90.5)
Defibrillator	37 (20.7)
Suction	62 (34.6)
**Drugs and IV fluids**	
Sublingual Nitrate	85 (47.5)
Glucagon	34 (19)
Aspirin	169 (94.4)
Furosemide	150 (83.8)
Morphine	29 (16.2)
Metoclopramide	147 (82.1)
Diazepam	130 (72.6)
Ventolin	167 (93.3)
Hyoscine	104 (58.1)
Normal saline	172 (96.1)
Ringer lactate	114 (63.7)
Dextrose 5%	126 (70.4)
Adrenaline injection	149 (83.2)
Antihistamine injection	153 (85.5)
Calcium chloride injection	20 (11.2)
Hydrocortisone injection	88 (49.2)
**Supporting Facilities**	
X ray	51 (28.5)
Laboratory	162 (90.5)
Equipped ambulance	11 (6.1)

Table 6 shows the primary healthcare physicians’ satisfaction regarding the management of emergency cases in their centers. The highest satisfaction rates were with managing severe dehydration (48%), followed by hypoglycemia (38.5%), anaphylaxis (33%) and acute asthma (30.7%). In contrast, physicians were the least satisfied with the management of cardiac arrest (26.8%), acute vaginal bleeding (21.8%), acute gastrointestinal bleeding (19%), myocardial infarction (18.4%), diabetic ketoacidosis (14.5%) and renal colic (14.5%).

**Table 6:** Level of satisfaction of primary health care physicians regarding emergency services provided at their primary health care centers

Emergency Cases	Frequency (%) *n* = 179				
	Not satisfied	Satisfied, but there’s a lack in equipment	Satisfied, but there’s a lack in medication	Satisfied, but there’s a lack in training	Satisfied
Acute asthma	10 (5.6)	45 (25.1)	46 (25.7)	23 (12.8)	55 (30.7)
Myocardial infarction	33 (18.4)	52 (29.1)	37 (20.7)	31 (17.3)	26 (14.5)
Angina pectoris	22 (12.3)	41 (22.9)	46 (25.7)	29 (16.2)	41 (22.9)
Cardiac arrest	48 (26.8)	47 (26.2)	14 (7.8)	47 (26.3)	23 (12.8)
Severe dehydration	11 (6.1)	33 (18.4)	32 (17.9)	17 (9.5)	86 (48)
Renal colic	26 (14.5)	31 (17.3)	43 (24)	32 (17.9)	47 (26.3)
Acute GI bleeding	34 (19)	61 (34.1)	25 (14)	36 (20.1)	23 (12.8)
Hypoglycemia	9 (5)	23 (12.8)	56 (31.3)	22 (12.3)	69 (38.5)
Diabetic ketoacidosis	26 (14.5)	31 (17.3)	43 (24)	32 (17.9)	47 (26.3)
Convulsion	18 (10.1)	24 (13.4)	46 (25.7)	42 (23.5)	49 (27.4)
Anaphylaxis	12 (6.7)	29 (16.2)	44 (24.6)	35 (19.6)	59 (33)
Acute vaginal bleeding	39 (21.8)	51 (28.5)	23 (12.8)	38 (21.2)	28 (15.6)

Concern about the lack of personnel training (physicians and nurses) was the greatest with cases of cardiac arrest (26.3%), convulsion (23.5%), acute vaginal bleeding (21.2%) and acute gastrointestinal bleeding (20.1%). Insufficient medications were a concern for managing hypoglycemia (31.3%), acute asthma (25.7%), angina pectoris (25.7%), diabetic ketoacidosis (24%) and renal colic (24%). Inadequate facilities and equipment were the greatest concern for addressing acute GI bleeding (34.1%), myocardial infarction (29.1%), acute vaginal bleeding (28.5%) and cardiac arrest (26.2%).

Table 7 presents the barriers to emergency case management. One-third of physicians (36.3%) indicated that the nonavailability of appropriate equipment was an issue. Lack of training in emergency management was a barrier for 24%.

**Table 7:** Barriers that prevent primary health care physicians from managing emergent cases

Cause	Frequency (%)
Patient arrived late and there is no time to transfer it to hospital	34 (19.0%)
Not enough training about managing emergent cases through the studying period	43 (24.0%)
Nonavailability of appropriate equipment required for managing an emergent case	65 (36.3%)
The clinic is crowded with patients making it hard to identify a silent emergency case.	37 (20.7%)

## Discussion

Preparation for emergency care management in the primary care setting is a global problem [2]. It is especially important in regions where travel is delayed or difficult, or hospitals are not easily accessible [1]. Given the current strife in Palestine, assessing the capability and preparedness of primary care centers and physicians to handle emergencies is timely.

Palestine has been occupied by Israel for decades with periods of increased conflict [14]. Due to years of inadequate investment in health facilities and personnel [14], it is not surprising that our research shows that the primary healthcare providers and centers in the West Bank are not adequately prepared to handle medical emergencies. The majority of the primary health care centers lacked appropriately furnished ambulances, and almost half of the centers did not have X-ray machines. Availability of supplies and medications was variable. Since the Gaza war has impacted imports and finances, the stocks and expenses of medications have grown worse since the time of our study [15].

General practitioners, who have not pursued specialization, provide most of the primary care in the health centers. For the most part, their training has not equipped them with the expertise and skills to handle emergency cases. However, a substantial percentage of them received training in BLS, ACLS, or ATLS courses within a one- and two-year time frame. Participation in these courses demonstrates a proactive approach to tackling the specific healthcare difficulties encountered in these high-risk areas, where the prompt and skilled delivery of emergency care is of utmost importance.

The specialty of family medicine started in 2011 in Palestine [16]. There is only one residency that has graduated 54 practicing family physicians, hardly enough to staff all primary health care centers. Their training includes both BLS and ACLS.

Other studies examining emergency preparedness in primary care echo our findings. Riyadh Saudi Arabia identified the lack of equipment as the main barrier [8].Studies conducted in Jeddah [3] and Spain [17] identified suboptimal training in managing emergency cases and not attending the emergency courses as the main barriers. Dammam Saudi Arabia found that two-thirds of PHC physicians reported good knowledge in the management of emergency cases, but 50–70% wanted further training in one or more of the major and common emergencies [18]. Physicians and organizations acknowledge the urgent necessity to improve the emergency abilities of physicians, particularly in high-risk regions [19].

The integration of efficient emergency care management into primary care helps to lessen the burden on hospital emergency rooms [18, 20–22]. It also lowers mortality and disability and can impact the entire health system [23, 24]. Preparedness involves equipped facilities, appropriate supplies and medications, and routine health care personnel training.

Improving the emergency preparedness of primary health centers requires resources and a comprehensive plan. In the future, we encourage the Ministry of Health to better equip primary health centers. Our research supports the continued investment in training programs like BLS, ACLS, and ATLS, to provide primary care physicians with the essential skills to effectively handle cardiac and other emergencies. In addition, evidence-based health-care guidelines are not routinely used in Palestine [25]. Including evidence-based guidelines in training or continuing education and adopting them at the ministry or professional association level, may improve the management of cases such as acute asthma, GI bleeding, and convulsions.

### Limitations

As a result of the ongoing conflict, the questionnaire was distributed online to physicians, resulting in a smaller-than-anticipated sample size likely due to unusually busy health center schedules, decreased days in the centers because of budget constraints, and internet disruptions. In addition, we were only able to conduct this study in the northern region of Palestine. Equipment and supplies in the centers were self-reported as was frequency of encountering types of cases. As Self-perceived competencies are inherently subjective and therefore require particular focus. So, An in-person center assessment and review of the center’s patient visits would have been more accurate, but impossible to do.

## Conclusion

The present study showed that emergency services at the primary health care level in northern areas of Palestine are functioning but have room for improvement. The level of training and emergency courses of primary health care physicians are suboptimal particularly in ATLS and ACLS courses. Depending on that, Create and implement extensive training programs for primary health care physicians, notably targeting emergency courses, and upgrade emergency equipment in primary health care clinics is recommended. To increase the quality of emergency treatment supplied, healthcare staff can be given specific training that improves their skills and knowledge. also A prospective study aims to assess patient satisfaction and perceptions of emergency services provided in primary healthcare facilities. This will lead to better patient outcomes.

Defects revealed by the present study, and made worse by the present conflict, will need to be planned for and addressed when resources become available in order to enhance the quality of the emergency services provided at primary health care centers in Palestine.

## Data Availability

The datasets used and/or analysed during the current study available from the corresponding author on reasonable request.

## Appendix 1

Questionnaire

**Table:**

**Sociodemograoic data**
1- Age
2- Gender
3- Marital status
4- Residency
**Level of training**
**Job**: o GP o Specialist If specialist what is the specialty ………….
**What board you Have?** o Palestinian o Arabic o Jordanian o Other
**Years of working in primary health care**: o < 1 year o 1–5 years o > 5 years
**Working experience in emergency department**: o < 1 year o 1–2 years o > 2 years

**Table:**

Have you ever attended?		If yes, last time you attended was
Basic life support (BLS)	o Yes o No	o < 1 year o 1–2 years o > 2 years
Advanced cardiac life support (ACLS)	o Yes o No	o < 1 year o 1–2 years o > 2 years
Advanced trauma life support (ATLS)	o Yes o No	o < 1 year o 1–2 years o > 2 years
If your answer in the previous 3 questions is **NO** please identify the most possible cause: o Not enough time o Loss of motivation o High cost o Availability of courses o Difficulty in obtaining educational leave from your job		

**Table:**

Frequency of emergency cases seen by PHC physicians			
**Number of cases you encountered in the last 12 months**	0	1–2	> 2
Acute asthma			
Myocardial infarction			
Angina pectoris			
Cardiac arrest			
Severe dehydration			
Renal colic			
Hypoglycemia			
Diabetic ketoacidosis			
Acute GI bleeding			
Acute vaginal bleeding			
Anaphylaxis			
Convulsion			

**Table:**

Physicians’ perceived competence when dealing with emergency cases				
Please put a tick in the place where you believe it reflects your capabilities in dealing with emergency cases				
	I do not know where to start	I will do only if no one else is available	I will attempt in most cases	I will attempt in all cases
Cardiac compression				
Mouth-mouth resuscitation				
Bag & mask resuscitation				
Nebulization & O2 therapy				
Inserting IV cannula				
Inserting urinary catheter				
Reading ECG				
Defibrillation				
Simple suture				

**Table:**

Availability of items needed for emergency care at PHC centers in Palestine		
Please put a tick ONLY if available		
□ Cannula □ Iv stand □ Urinary catheter □ Dressing tray □ Side lamp with stand □ Splint □ Scissors □ Suture material □ Ambu bag □ Nebulizer □ O2 cylinder with standard fit □ Airway equipment □ Oxygen mask □ Defibrillator □ Suction apparatus	**Drugs and IV fluid** □ Sublingual nitrate □ Glucagon □ Aspirin □ Furosemide □ Morphine □ Metoclopramide □ Diazepam □ Ventolin □ Hyoscine □ Normal saline □ Ringer lactate □ Dextrose 5%,10%,50% □ Adrenaline injection □ Antihistamine injection □ Calcium chloride injection □ Hydrocortisone injection	**Supporting facilities**: □ X ray □ Laboratory □ Equipped ambulance car

**Table:**

The most frequent barrier preventing you from managing an emergent case is (choose 1 only)
□ Lack of time: -for example Difficulty in reaching the hospital.
□ Lack of exposure to emergent cases.
□ Lack of equipment.
□ Busy clinic. □ Other cause. -If you answered with other reasons, please specify the reason:

**Table:**

Satisfaction of PHC physicians regarding the emergency services provided at their PHC centers					
	1	2	3	4	5
Acute asthma					
Myocardial infarction					
Angina pectoris					
Cardiac arrest					
Severe dehydration					
Renal colic					
Hypoglycemia					
Diabetic ketoacidosis					
Acute GI bleeding					
Acute vaginal bleeding					
Anaphylaxis					
Convulsion					

- 1: Not satisfied.
- 2: Satisfied but equipment are deficient.
- 3: Satisfied but medications are deficient.
- 4: Satisfied but we need more training for staff.
- 5: fully satisfied.
